# Role of TNF-α in Regulating the Exercise Pressor Reflex in Rats With Femoral Artery Occlusion

**DOI:** 10.3389/fphys.2018.01461

**Published:** 2018-10-15

**Authors:** Jihong Xing, Jian Lu, Jianhua Li

**Affiliations:** ^1^Heart & Vascular Institute, The Pennsylvania State University College of Medicine, Hershey, PA, United States; ^2^Department of Emergency Medicine, The First Hospital of Jilin University, Changchun, China

**Keywords:** muscle afferent nerve, TNF-α, peripheral arterial disease, hindlimb ischemia, exercise, blood pressure

## Abstract

Responses of sympathetic nerve activity and arterial blood pressure are augmented during activation of the exercise pressor reflex in rats with femoral artery occlusion. The present study examined the role played by proinflammatory tumor necrosis factor-α (TNF-α) in regulating augmented sympathetic responsiveness induced by stimulation of muscle metabolic receptors and static muscle contraction following 72 h of femoral artery occlusion. We first observed that the levels of TNF-α and protein expression of TNF-α receptor type 1 (TNFR1) were increased in the dorsal root ganglion (DRG) of hindlimbs with femoral artery occlusion. Note that TNF-α was observed within DRG neurons of C-fiber afferent nerves. Capsaicin (TRPV1 agonist) and AITC (TRPA1 agonist) were injected into arterial blood supply of the hindlimbs to stimulate metabolically sensitive thin-fiber muscle afferents. The effects of these injections on the sympathetic and pressor responses were further examined in control rats and rats with femoral artery occlusion. As TNF-α synthesis suppressor pentoxifylline (PTX) was previously administered into the hindlimb with femoral artery occlusion, sympathetic, and pressor responses induced by capsaicin and AITC were attenuated. In occluded rats, PTX also attenuated the exaggeration of blood pressure response induced by muscle contraction, but not by passive tendon stretch. Overall, the results suggest that TNF-α plays a role in modulating exaggerated sympathetic nervous activity via the metabolic component of the exercise pressor reflex when the hindlimb muscles are ischemic in peripheral arterial disease.

## Introduction

Exercise activity increases sympathetic nervous activity (SNA) and decreases parasympathetic activity and thereby amplifies blood pressure (BP) and heart rate (HR), myocardial contractility and peripheral vasoconstriction (Mitchell et al., 1983). A mechanism termed the “Exercise Pressor Reflex” contributes to sympathetic engagement during exercise (McCloskey and Mitchell, 1972; Mitchell et al., 1983). This autonomic reflex is evoked when thin fiber afferents arising from contracting skeletal muscle are activated (Kaufman and Forster, 1996). Mechanical deformation of the muscle afferents receptive field and muscle metabolic by-products are involved in this reflex responses (Kaufman and Forster, 1996). When thin fiber muscle afferent nerves are stimulated, cardiovascular nuclei in the brainstem are activated and SNA, BP, and HR are increased (Mitchell et al., 1983).

Mechanically sensitive Group III afferents and chemically sensitive Group IV afferents are primarily engaged in the exercise pressor reflex (Kaufman et al., 1984b). Group III afferent fibers frequently demonstrate mechanical sensitivity and contribute to the autonomic adjustments to exercise. For example, group III afferents often respond vigorously at the onset of contraction, with their first impulse being discharged with a latency of 200 ms or less (Kaufman et al., 1983). Moreover, group III afferents increase their responses to contraction as the tension developed by the working muscle increases (Kaufman et al., 1983; Mense and Stahnke, 1983; Hayward et al., 1991). Group IV afferents possess different discharge properties than do group III afferents. Although both respond to contraction, group IV afferents do not discharge vigorously at the onset of contraction. Instead, group IV afferents usually respond to contraction with latencies of 5–30 s (Kaufman et al., 1983; Mense and Stahnke, 1983). In addition, about half of the group IV afferents respond more to a static contraction when the contraction is performed ischemically and not under freely perfused conditions (Bessou and Laporte, 1958; Iggo, 1961; Mense and Stahnke, 1983; Kaufman et al., 1984a). Moreover, intra-arterial injection of muscle metabolites has been shown to stimulate at least half of the group IV afferents tested (Hník et al., 1969; Kumazawa and Mizumura, 1977; Mense, 1977; Rybicki et al., 1985; Thimm and Baum, 1987). Intra-arterial injection of chemicals (i.e., capsaicin and AITC) stimulating metabosensitive transient receptor potential vanilloid receptor 1(TRPV1) and transient receptor potential ankyrin 1 (TRPA1) in muscle afferent nerves also increases reflex sympathetic and BP responses (Xing et al., 2009, 2015). Thus, in the current study, we stimulated TRPV1 and TRPA1 and examined their effects on SNA and BP responses.

With an atherosclerotic tissue peripheral arterial disease (PAD) decreases blood flow to the arteries of the lower extremities. Intermittent claudication is the most common symptom in PAD, and this is worsened by intense exercise as a result of muscle ischemia; however, this symptom can subside at rest with a lower metabolic demand of the muscles (Rejeski et al., 2008). During walking, a greater increase in arterial BP is observed in the PAD patients as compared with the normal subjects (Baccelli et al., 1997). Additionally, the exercise pressor reflex contributes to the exaggerated BP response to walking in PAD patients (Baccelli et al., 1999). In a rat with femoral artery occlusion, the SNA and BP responses are also augmented during muscle contraction or stimulation of muscle metabolic receptors (Tsuchimochi et al., 2010; Li and Xing, 2012).

The augmented exercise pressor reflex might be due to in part to inflammation, specifically pro-inflammatory cytokines (PICs), associated with PAD. Numerous cells (i.e., leukocytes, myocytes, microglia, astrocytes, and Schwann cells) produce and release PICs (Miller et al., 2009), which include interleukins, lymphokines, and cell signaling molecules. Especially, the roles of tumor necrosis factor-α (TNF-α), interleukin-6 (IL-6), and interleukin-1β (IL-1β) are significant in regulating immune and inflammatory reactions. These PICs modulate the activities of many cell types in various diseases. For example, during diseased states, PICs help to recruit cells to inflammatory sites, stimulating cell survival, division, and enhancing proliferation and differentiation (Oppenheim, 2001). Evidence indicates that PICs are involved in regulating physiological functions with their levels increasing in the circulation and in the affected tissues (Jonsdottir et al., 2000; Steensberg et al., 2000; Miller et al., 2009). Increased circulating and intramuscular levels of PICs (such as IL-6 and TNF-α) were also found in coronary and/or atherosclerotic vascular disorders such as PAD (Mann, 2002; Yndestad et al., 2006; El-Menyar, 2008).

Tetrodotoxin (TTX)-resistant Na^+^ (i.e., Na_V_1.8) channels are highly expressed in group IV afferents (Lai et al., 2004). The role played by Na_V_1.8 in evoking the exercise pressor reflex was examined using the whole animal preparations. A803467, a Na_V_1.8 blocker, attenuates the pressor response evoked by arterial injection of lactic acid and capsaicin stimulating thin fiber afferents (Stone et al., 2013). There is a linkage between TNF-α and activity of Na^+^ current in sensory nerves (Chen et al., 2011). A prior study demonstrated the role of TNF-α in enhancing the current densities of Nav1.8 in dorsal root ganglion (DRG) neurons (Chen et al., 2011).

Accordingly, the emphasis of the current study was to determine the role for TNF-α in engagement of the augmented sympathetic responsiveness induced by the exercise pressor reflex. We hypothesized that femoral artery occlusion increases TNF-α in the DRG and inhibition of TNF-α production in the hindlimb muscles with femoral artery occlusion alleviates overactive sympathetic and pressor responses induced by stimulation of metabolically sensitive muscle afferents nerves. Our general hypothesis was that TNF-α contributes to the exaggerated SNA induced by muscle contraction via the metabolic component of the exercise pressor reflex when arterial blood supply to the hindlimb muscle is deficient in PAD.

## Materials and Methods

### Ethical Approval

All animal experimental procedures were approved by *the Institutional Animal Care and Use Committee* of Pennsylvania State College of Medicine and complied with the National Institutes of Health guidelines.

### Femoral Artery Occlusion

Male Sprague–Dawley rats (250–300 g) were anesthetized with an isoflurane–oxygen mixture (2–5% isoflurane in 100% oxygen). For the immunofluorescence and Western blotting experiments, the femoral artery on one limb was surgically exposed, dissected, and ligated ∼3 mm distal to the inguinal ligament as previously described (Lu et al., 2013; Xing et al., 2015). In control, the same procedures were performed on the other side of limbs of the same rat except that a suture was placed below the femoral artery but was not tied. The limbs in which the femoral artery was ligated served as “ischemic limbs”; and the other side of limbs in the same rats served as “control limbs.” For the experiment using the whole animals, the rats were divided between those that had the right femoral artery occlusion (“occluded rats”) and the different rats that had sham surgeries on the right hindlimb (“control rats”). In another group, PTX (10 mg/kg daily for 3 days) was administered into the hindlimb muscles of the ischemic limbs of rats following ligation surgery. This approach was likely to inhibit TNF-α formation to a greater degree after femoral artery occlusion and before the experiment was performed. Seventy-two hours were allowed for recovery before the experiments began.

### ELISA Measurement

We analyzed the levels of TNF-α in the DRGs instead of its levels in the circulation since TNF-α in sensory nerve system closely indicate its effects on the exercise pressor reflex evoked by activation of muscle afferent nerves. The DRGs (L4–L6) of control limbs (*n* = 6) and ischemic limbs (*n* = 14) were removed after the rats were anesthetized and decapitation. Total protein was extracted by homogenizing DRG samples in ice-cold radioimmunoprecipitation assay buffer with protease inhibitor cocktail kit. The lysates were then centrifuged for 15 min at 4°C, and the supernatants were collected for measurements of protein concentrations using a bicinchoninic acid assay reagent kit. Briefly, polystyrene 96-well immunoplates were coated with affinity-purified polyclonal goat anti-TNF-α antibody (Signosis) and incubated overnight. Then, the diluted samples and TNF-α standard solution were distributed in each plate and incubated for 1 h at room temperature. After incubation, the plates were washed and then incubated with streptavidin-conjugated HRP for 45 min at room temperature. Then, substrate solution (100 μl) was added to each well and incubated for 30 min. The optical density was measured using an ELISA reader (BioTek).

### Fluorescence Immunohistochemistry

Three rats without femoral artery occlusion were anesthetized, and then transcardially perfused with 200 ml of ice-cold saline (containing 1,000 units heparin) followed by 500 ml of 4% ice-cold paraformaldehyde in phosphate-buffered saline (PBS). DRGs (L4–6) were dissected out and immersed in the same fixative at 4°C for 2 h. The tissues were then stored in PBS containing 30% sucrose overnight. Then, 10 μm of DRG sections were obtained using a cryostat.

DRG sections were fixed in 4% of paraformaldehyde in PBS for 10 min at room temperature. After being washed with PBS, the tissue were permeabilized, blocked in 0.3% Triton X-100 in PBS supplemented with 5% goat serum for 1 h, and then incubated with rabbit anti-TNF-α antibody (1:200, Novus; Cat#: NBP1-19532) overnight at 4°C. After being washed in PBS, the sections were incubated with goat anti-rabbit fluorescein isothiocyanate (FITC) labeled secondary antibody (1: 200, Invitrogen) for 2 h at room temperature.

To examine TNF-α within DRG neurons of C-fiber and/or A-fiber, the sections were incubated with the second primary antibody [mouse anti-peripherin (Cat#: ab17999) to label C-fiber at 1:200; and anti-NF200 (Cat#: ab19386) to label A-fiber at 1:200, Abcam)] overnight. After the sections were washed and incubated at room temperature with secondary antibody (Alexa Fluor-594 conjugated goat anti-mouse IgG at 1:200), they were cover slipped. A Nikon Eclipse 80i microscope was used to examine FITC- and Alexa Fluor-594-labeled DRG neurons, and the images were stored digitally on a computer.

### Western Blot Analysis

We used six rats to examine expression of TNF-α receptor type 1 and 2 (TNFR1 and TNFR2) in the L4–6 DRGs of control limbs and ischemic limbs. Western blot was performed as previously described (Liu et al., 2010). In brief, all DRGs from respective rats were removed and sampled. Total protein was extracted by homogenizing DRG samples, and the lysates were centrifuged. The supernatants were then collected to determine protein concentrations.

After being denatured by heating at 95°C for 5 min in an SDS sample buffer, the supernatant samples with 20 μg of protein was loaded onto 4–20% Mini-Protean TGX Precast gels (Bio-Rad Lab) and then electrically transferred to a polyvinylidene fluoride membrane. The membrane was blocked by 5% nonfat milk in 0.1% Tween-TBS buffer for 1 h and was then incubated overnight with primary antibodies (1:500): rabbit anit-TNFR1 (Cat#: Ab19139) and rabbit anit-TNFR2 (Cat# Ab109322).

Then, the membrane was incubated with horseradish peroxidase-linked anti-rabbit secondary antibody (1:1000, Abcam) and visualized for immunoreactivity using an enhanced chemiluminescence system (Cell Signaling Tech). The membrane was stripped and incubated with anti-β-actin. The densities of TNFR1/TNFR2 and β-actin bands were examined using the NIH Scion Image Software.

### Examination of Sympathetic and BP Responses

The rats were anesthetized with a mixture of 2–5% isoflurane and oxygen and ventilated as described previously (Xing et al., 2015). The jugular vein and common carotid artery were cannulated to deliver fluids and to connect a pressure transducer for measurement arterial BP. HR was calculated on a basis of beat to beat from the arterial pressure pulse and also recorded. A catheter (PE10) was then inserted into the femoral artery for injection of drugs. During the experiments, baseline BP and fluid balance were maintained with a continuous infusion of saline. Body temperature was continuously monitored and maintained at 37.5–38.5°C with a heating pad and external heating lamps (Xing et al., 2015).

A bundle of the renal nerves on the left side was dissected from other connective tissues. A piece of laboratory film was placed under the isolated nerves, and two tips of a bipolar electrode for recording neural activity were placed between the nerves and the film; these were embedded in a silicone gel. Once the gel hardened, the silicone rubber was fixed to the surrounding tissue. The renal SNA (RSNA) signal was amplified with an amplifier (P511, Grass Instruments) with a band-pass filter of 300 Hz in low-cut frequency and of 3 kHz in high-cut frequency and recorded as previously described (Koba et al., 2008).

Decerebration was performed in order to minimize the effects of anesthesia on the reflex pressor response. Prior to the procedure, dexamethasone (0.2 mg, i.v.) was injected to minimize brain stem edema. A transverse section was made anterior to the superior colliculus and extending ventrally to the mammillary bodies. All tissues from rostral to the section were removed. Following this procedure, the anesthesia was withdrawn from the rats and rats were on a ventilator, and 60 min were allowed before the experiment began.

In the first group of experiments, the effects of TNF-α on RSNA and BP responses to activation of the metabolically sensitive TRPV1 and TRPA1 receptors were examined. In order to stimulate those receptors, TRPV1 agonist, capsaicin (0.25, 0.5, 1.0 μg/kg); and TRPA1 agonist, AITC (20, 40, 60 μg/kg) were injected into arterial blood supply of hindlimb muscles of control rats (*n* = 18), occluded rats (*n* = 14), and occluded rats with prior TNF-α synthesis suppressor pentoxifylline (PTX, *n* = 16). Capsaicin and AITC were given in random order. PTX (10 mg/kg/day for 3 days) was previously administered into the hindlimb muscles following femoral artery occlusion. The concentrations of capsaicin and AITC were selected on the basis of the results of the prior studies (Xing et al., 2008, 2015). The injection volume was 0.1–0.2 ml according to rat’s body weight. The duration of the injection was 1 and 20 min were allowed between injections.

In the second group of experiments, a laminectomy was performed to expose the lower lumbar and upper sacral portions of the spinal cord and peripheral ends of the transected L4 and L5 ventral roots were placed on platinum bipolar stimulating electrodes (Smith et al., 2001). Static muscle contractions were performed by electrical stimulation of the L4 and L5 ventral roots (30 s, three-times motor threshold with a period of 0.1 ms at 40 Hz). The reflex BP and HR responses to contraction were examined in control rats (*n* = 8), occluded rats (*n* = 10), and occluded rats with prior injection of PTX (*n* = 8). This experiment was to examine if inhibition of TNF-α altered the effects of femoral artery occlusion on the pressor responses evoked by muscle contraction (stimulation of the mechanical and metabolic components of the exercise pressor reflex). Although the muscle mechanoreflex component of the exercise pressor reflex is not largely involved in its overactivity in occluded rats, it was unclear if TNF-α can alter muscle metabolites in ischemic muscles and affect this component of the reflex. Thus, in order to activate the mechanical component of the exercise pressor reflex the passive tendon stretch was performed in the right hindlimb of control rats (*n* = 8), occluded rats (*n* = 6), and occluded rats with PTX (*n* = 6). Passive tendon stretch (500 g of tension) was produced manually over ∼5 s by using a rack and pinion attached to the Achilles’ tendon of rats. Each bout of muscle stretch was maintained for 30 s after 500 g of tension was achieved.

### Experimental Data and Statistical Analysis

Mean arterial pressure (MAP) was obtained by integrating the arterial signal with a time constant of 4 s. HR was calculated on a basis of beat to beat from the arterial pressure pulse. The peak responses of MAP and HR were determined by the peak change from the control value. RSNA signals were transformed into absolute values, integrated over 1 s interval, and subtracted by 1 s of integrated background noise. To quantify RSNA response to capsaicin and AITC injection, baseline values were obtained by taking the mean value for the 30 s immediately before each injection and by ascribing the mean value of 100%, and relative change from baseline during the injection were then evaluated.

Two-way repeated measures analysis of variance (ANOVA) was used to analyze experimental data with Tukey’s *post hoc* tests as appropriate. All values were presented as mean ± SEM. SPSS for Windows version 21.0 was used for all statistical analyses, and differences were considered significant at *P* < 0.05.

## Results

### Levels of TNF-α

**FIGURE 1:**
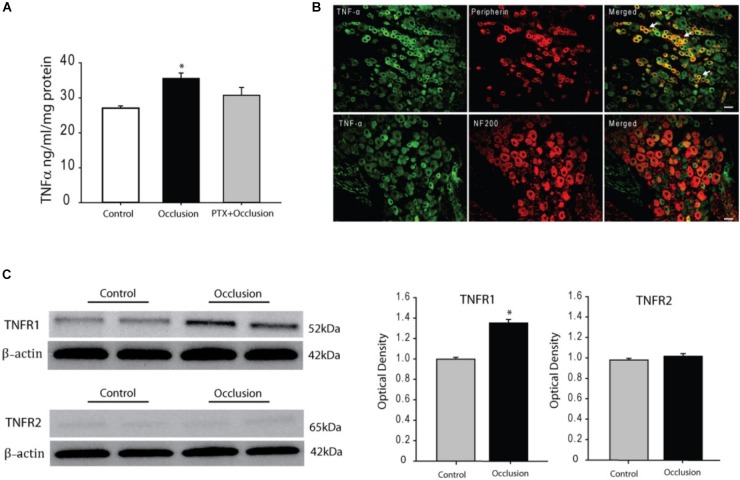
Effects of femoral artery occlusion on TNF-α signal in sensory nerves. **(A)** Seventy-two hours of femoral artery occlusion increased the levels of TNF-α in the DRG tissues as compared with them in DRG of control limbs (*n* = 6 in each group); and PTX given into the hindlimb muscles attenuated amplification of TNF-α in the DRG tissues of limbs with femoral artery occlusion (*n* = 8). ^∗^*P* < 0.05, occlusion group vs. control and occlusion with prior PTX. **(B)** Immunofluorescence was used to examine double-labeling for TNF-α and peripherin/NF-200 (*n* = 3). Peripherin was used to label DRG neurons that project thin C-fibers. NF200 was used to identify A-fibers of DRG neurons. Representative photomicrographs show co-existence of TNF-α and peripherin staining in DRG neurons (top panel), whereas few TNF-α and NF-200 staining were observed in DRG neurons (bottom panel). Arrows indicate representative positive cells for both TNF-α and peripherin after they were merged. Scale bar = 50 μm. **(C)** Representative bands (left panel) and averaged data (right panel), demonstrating that femoral artery occlusion upregulated protein expression TNF-α receptor subtype TNFR1, but not TNFR2. A significant difference in TNFR1 was seen between control and occluded groups. ^∗^*P* < 0.05 vs. control. *n* = 6 in each group.

Seventy-two hours of femoral artery occlusion significantly increased the levels of TNF-α in the DRG tissues of ischemic limbs as compare with control limbs (*n* = 6 in each group). Thus, 72 h of femoral artery occlusion was used for other experiments in this report. In another group, we further determined the effects of PTX treatment on TNF-α level 72 h after femoral artery occlusion (*n* = 8). **Figure 1A** shows that TNF-α was amplified in the DRG by femoral artery occlusion and PTX administered previously into the hindlimb muscles attenuated increases of TNF-α in the DRG induced by 72 h of femoral artery occlusion (*P* < 0.05, occlusion vs. control and occlusion with PTX treatment).

### TNF-α Within DRG Neurons With Different Fiber Types

We also examined if TNF-α exists within DRG neurons projecting C- and/or A-fiber afferents. Peripherin was used to label DRG neurons that project thin C-fibers. NF200 was used to identify A-fibers of DRG neurons. The dual immunofluorescence techniques were employed to determine co-localization of fluorescent TNF-α and peripherin/NF200 immunoreactivity in the DRG neurons of rats without femoral artery occlusion (*n* = 3). The appearance of TNF-α and peripherin/NF200 within DRG neurons is characterized by fluorescent green and red color, respectively (**Figure 1B**). The photomicrographs show that TNF-α largely appears within C-fiber of DRG neurons, but TNF-α barely is seen within A-fiber of DRG neurons.

### Protein Levels of TNFR1 and TNFR2

The protein expression of TNF-α receptors, namely TNFR1 and TNFR2, in the DRG tissues of control limbs and ischemic limbs was examined. Typical bands and average data of **Figure 1C** show that TNFR1 expression was significantly increased 72 h after femoral artery occlusion. The density of the signal in the DRG tissues of ischemic limbs was ∼1.36-fold greater than that in control limbs (optical density: 1.35 ± 0.04 in occlusion vs. 0.99 ± 0.02 in control, *P* < 0.05; *n* = 6 in each group). **Figure 1C** also demonstrates that no differences were observed in the expression of TNFR2 in the DRG tissues of control limbs and ischemic limbs.

### RSNA and BP Responses to Activation of TRPV1 and TRPA1

**FIGURE 2:**
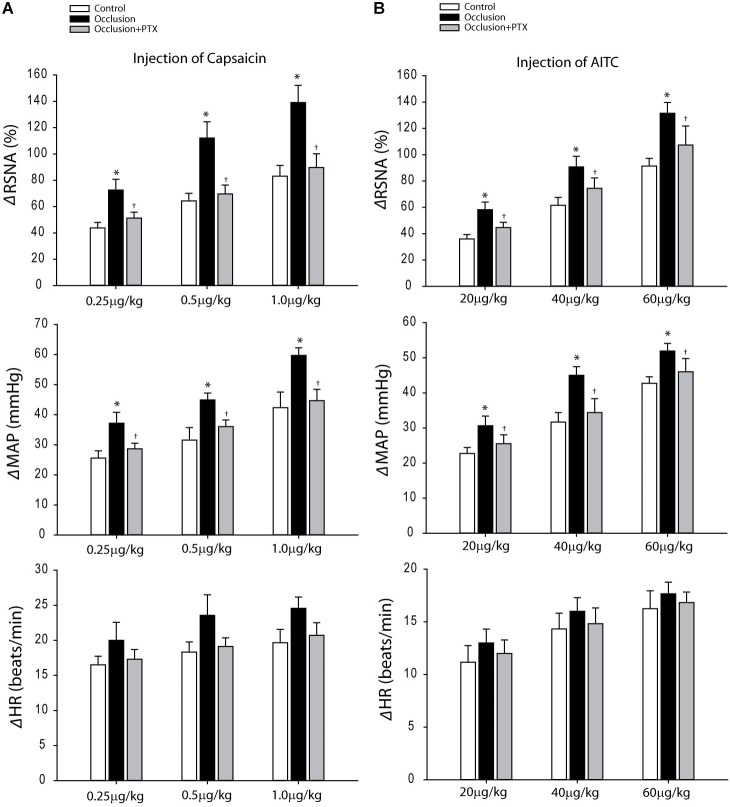
Responses of RSNA, MAP, and HR evoked by stimulation of TRPV1 and TRPA1 receptors with arterial injection of capsaicin and AITC. **(A)** Three dosages of capsaicin evoked increases in RSNA and MAP in a dose-dependent manner in control rats (*n* = 10) and occluded rats (*n* = 8). The effects were greater in occluded rats. As PTX was given previously in occluded rats (*n* = 8), amplified RSNA and MAP responses were attenuated. ^∗^*P* < 0.05, compared with control rats and ^†^*P* < 0.05, compared with occluded rats without PTX. **(B)** In the similar way, the RSNA and MAP were increased after injection of three dosages of AITC in control rats (*n* = 8), occluded rats (*n* = 6), and occluded rats with PTX treatment (*n* = 8). The responses were enhanced in occluded rats and PTX can attenuate increases of RSNA and MAP responses. ^∗^*P* < 0.05, compared with control rats and ^†^*P* < 0.05, compared with occluded rats without PTX.

Baseline values for MAPs and HRs before arterial injections of capsaicin are 90 ± 4 mmHg and 388 ± 22 bpm in control rats (*n* = 10); 91 ± 3 mmHg and 383 ± 18 bpm in occluded rats (*n* = 8); and 93 ± 5 mmHg and 386 ± 22 bpm in occluded rats with PTX (*n* = 8). No significant differences in basal MAP and HR were observed before injections among three groups (*P* > 0.05 among groups). **Figure 2A** demonstrates that femoral artery occlusion amplified the responses of RSNA and MAP evoked by capsaicin (0.25, 0.5, and 1.0 μg/kg). This figure also shows amplified RSNA and MAP responses to capsaicin in occluded rats were attenuated after inhibition of TNF-α with PTX, that is, when 0.25 μg/kg capsaicin was given, the RSNA and MAP responses were 44 ± 4% and 26 ± 2 mmHg in control rats; 73 ± 8% and 37 ± 4 mmHg in occluded rats; and 51 ± 5% and 29 ± 2 mmHg in occluded rats with PTX treatment (*P* < 0.05, occlusion vs. control and PTX treatment).

Baseline MAPs and HRs were 89 ± 4 mmHg; 392 ± 23 bpm in control rats (*n* = 8) and 92 ± 2 mmHg; 385 ± 15 bpm in occluded rats (*n* = 6) and 92 ± 4 mmHg; 391 ± 21 bpm in occluded rats with PTX (*n* = 8) before injection of AITC (*P* > 0.05, among groups). **Figures 2B** demonstrates that femoral artery occlusion significantly increased the responses of RSNA and MAP evoked by 20, 40, and 60 μg/kg of AITC. Also, this figure shows RSNA and MAP responses to arterial injection AITC after PTX. PTX attenuated amplification of RSNA and MAP responses induced by injection AITC, that is, with 40 μg/kg AITC, the RSNA and MAP responses were 62 ± 6% and 32 ± 3 mmHg in control rats; 91 ± 8% and 45 ± 2 mmHg in occluded rats; and 75 ± 8% and 34 ± 4 mmHg in occluded rats with PTX treatment (*P* < 0.05, occlusion vs. control and PTX treatment).

### BP Response to Muscle Contraction and Tendon Stretch

**FIGURE 3:**
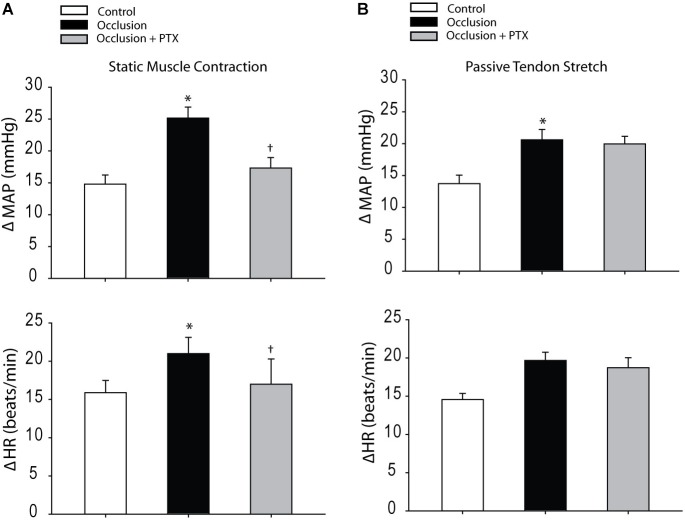
Responses of MAP and HR evoked by static muscle contraction and passive tendon stretch. **(A)** Muscle contraction was evoked by electrical stimulation of the L4 and L5 ventral roots. Femoral artery occlusion enhanced MAP and HR responses as compared with control rats. In occluded rats (*n* = 10), PTX significantly inhibited amplification of MAP responses induced by muscle contraction. ^∗^*P* < 0.05, compared with control rats and ^†^*P* < 0.05, compared with occluded rats without PTX (*n* = 8 in each group). No significant differences were seen in muscle developed tension among three groups. For example, 535 ± 30 g in control, 547 ± 28 g in occluded rats, and 550 ± 35 g in occluded rats with PTX. **(B)** Showing that femoral artery occlusion amplified MAP response induced by the passive tendon stretch, but PTX failed to attenuate amplification of MAP response (*P* > 0.05, occlusion vs. occlusion plus PTX; *n* = 6 in each group). Muscle tension was ∼500 g. ^∗^*P* < 0.05, compared with control rats (*n* = 8).

Baseline MAPs and HRs were 96 ± 4 mmHg, 379 ± 20 bpm in control rats (*n* = 8); 93 ± 5 mmHg, 377 ± 20 bpm in occluded rats (*n* = 10) and 92 ± 5 mmHg, 376 ± 19 bpm in occluded rats with injection of PTX (*n* = 8) (*P* > 0.05, among groups for MAP and HR). **Figure 3A** demonstrates that femoral artery occlusion significantly increased MAP and HR responses evoked by muscle contraction. Also, this figure illustrates MAP and HR responses to muscle contraction after inhibition of TNF-α with injection of PTX in occluded rats. PTX inhibited increase of MAP response induced by muscle contraction in occluded animals. The MAP responses were 25 ± 2 mmHg in occluded rats and 17 ± 2 mmHg in occluded rats with PTX treatment (*P* < 0.05, occlusion vs. occlusion plus PTX). Note that there were no significant differences in muscle developed tension among three groups. Furthermore, **Figure 3B** shows that femoral artery occlusion amplified MAP response induced by passive tendon stretch, but PTX failed to attenuate amplification of MAP response (*P* > 0.05, occlusion vs. occlusion plus PTX).

## Discussion

The current study examined the role played by TNF-α in augmented sympathetic responsiveness during activation of the exercise pressor reflex following 72 h of femoral artery occlusion. Our data showed that TNF-α was increased in the DRG of limbs with occluded femoral arteries and that TNF-α was present in DRG neurons supplying C-fiber afferents. Femoral artery occlusion further upregulated protein expression of TNF-α subtype receptor TNFR1. Blocking production of TNF-α by prior administration of PTX in ischemic limbs significantly attenuated enhancement of sympathetic and BP responses induced by stimulation of metabolic receptors of thin fiber muscle afferent nerves, as well as by static muscle contraction, but not by muscle stretch. These data suggest that TNF-α plays a role in modulating augmented sympathetic and BP responses in occluded femoral arteries via the metabolic component of the exercise pressor reflex.

Previous studies have demonstrated that inflammation contributes to the development of intermittent claudication in PAD by adversely affecting skeletal muscle function (Pande et al., 2015). Elevated inflammatory biomarkers (i.e., IL-6 and TNF-α) are inversely associated with physical performance in patients. For example, increases of IL-6 and TNF-α in PAD induce skeletal muscle protein breakdown and they are negatively related to muscle mass and strength (Goodman, 1991, 1994; Visser et al., 2002; Signorelli et al., 2003). Also, in large patient populations, elevated inflammatory biomarkers are associated with the incidence and severity of PAD (Pradhan et al., 2002, 2008; McDermott et al., 2003). Monocyte mRNA expression of TNF-α is associated with maximal walking time in patients with intermittent claudication (Pande et al., 2015). Those prior studies also suggest that circulating levels of PICs (such as IL-6 and TNF-α) are considerably elevated in populations of patients with PAD and correlate with impaired maximal walking time. In this report, the role for TNF-α in regulating the augmented sympathetic responsiveness induced by the exercise pressor reflex in PAD was emphasized. We observed co-localization of TNF-α and peripherin in DRG neurons and further found that TNF-α in the DRG was increased by femoral artery occlusion. It is speculated that amplification of TNF-α was likely to increase C-fiber afferent-mediated sympathetic and pressor responses as the hindlimb muscles were ischemic.

TNF-α plays a role by stimulating TNF-α receptor subtypes, TNFR1 and TNFR2 (MacEwan, 2002). TNFR1 is expressed on neuronal cells and plays a functional role, whereas TNFR2 is located primarily on macrophages and/or monocytes in response to inflammation. TNF-α activation appears most relevant to the development of afferent nerve-mediated behavior via the TNFR1. Mechanical hyperalgesia induced by exogenous TNF-α and/or by inflammation is attenuated in TNFR1 knockout mice; and TNFR1 (but not TNFR2) neutralizing antibodies attenuate experimental hyperalgesia (Sommer et al., 1998; Parada et al., 2003). Consistently, we observed a greater expression of TNFR1 in the DRG of limbs with femoral artery occlusion, suggesting the engagement of TNFR1 in the amplified exercise pressor reflex.

PTX is a phosphodiesterase inhibitor found in small clinical trials by blocking cytokine expression (Sliwa et al., 2002). Besides elevation of intracellular cAMP, PTX depresses production of TNF-α (Sliwa et al., 2004). In this report, PTX was used to block TNF-α in ischemic hindlimbs, and our data showed the contribution of TNF-α to amplify the exercise pressor reflex in rats with occluded femoral arteries.

The mechanisms by which increased TNF-α enhances the exercise pressor reflex are likely associated with changes in channels on muscle afferent nerves. A previous study demonstrated that TNF-α increased the current densities of Nav1.8 in DRG neurons (Chen et al., 2011). A blockade of Nav1.8 in muscle afferent nerves by its antagonist can attenuate the pressor response evoked by arterial injection of lactic acid and capsaicin (Stone et al., 2013). Inhibitory effects of TNF-α on K^+^ current in sensory neurons (Liu et al., 2008; Chen et al., 2011) are also likely involved in the augmented exercise pressor reflex in rats with femoral artery occlusion.

TRPV1 and TRPA1 in muscle afferent nerves are also likely a part of the mechanism leading to the enhanced exercise pressor reflex by activation of TNF-α. TRPV1 and TRPA1 are present in sensory nerves and respond to chemical stimuli in the processing of inflammation (Basso and Altier, 2017). It should be noted that inhibition of TNF-α has been reported to decrease expression of TRPV1 and/or TRPA1 and attenuate the effects of these receptors’ activation in sensory nerves in the processing of inflammatory responses (Hu et al., 2010; El Karim et al., 2015; Meng et al., 2016). In the prior studies, we have observed that upregulated TRPV1 and TRPA1 in the DRG of ischemic hindlimbs enhances the reflex sympathetic and BP responses in rats (Xing et al., 2008; Xing et al., 2015). Blocking TRPA1 also attenuates the exaggerated exercise pressor reflex in occluded rats (Xing et al., 2015). Interestingly, in this report, with the prior PTX amplified SNA and BP responses evoked by stimulation of individual TRPV1 and TRPA1 were significantly attenuated in rats with occluded femoral arteries. It is speculated that upregulated TRPV1 and/or TRPA1 in sensory nerves of ischemic hindlimbs are likely decreased by chronic administration of PTX. This suggests that TNF-α is a part of signal pathways engaged in the metabolic component of the reflex.

In addition, we examined BP response evoked by static muscle contraction and passive tendon stretch. Muscle contraction was performed to simultaneously activate muscle mechanoreflex and metaboreflex whereas passive stretch was used to activate the mechanoreflex only. Our data demonstrated that prior administration of PTX attenuated amplification of BP response by muscle contraction, but not by tendon stretch, further suggesting involvement of TNF-α in the metabolic component of the exercise pressor reflex.

### Study limitations

A possibility cannot be ruled that TNF-α was increased in the glial cells. In addition, TNFR1 in the DRG tissues was examined by Western blot analysis. Using this method, it was unlikely to determine if the upregulation of TNFR1 of the PAD rats was specific in C-fibers.

## Conclusion

Femoral artery occlusion amplifies the levels of TNF-α in the DRG and thereby results in heightened expression of TNFR1. We further observed that increased TNF-α is present within DRG neurons of C-fiber afferent nerves. Moreover, inhibition of TNF-α production in hindlimb muscles with femoral artery occlusion alleviates exaggeration of SNA and pressor responses during stimulation of metabolically sensitive muscle afferents nerves and during activation of the metabolic component of the exercise pressor reflex.

## Author Contributions

JX and JLi participated in the design of the experiment and drafting the manuscripts. JX and JLu contributed to the data collection and analysis. JLi contributed to revising the article critically for important intellectual content. All authors approved the final version.

## Conflict of Interest Statement

The authors declare that the research was conducted in the absence of any commercial or financial relationships that could be construed as a potential conflict of interest.
